# The role of prolactin/vasoinhibins in cardiovascular diseases

**DOI:** 10.1002/ame2.12264

**Published:** 2022-08-03

**Authors:** Hui Zhao, Sugang Gong, Yongcong Shi, Cijun Luo, Hongling Qiu, Jing He, Yuanyuan Sun, Yuxia Huang, Shang Wang, Yuqing Miao, Wenhui Wu

**Affiliations:** ^1^ School of Materials and Chemistry & Institute of Bismuth and Rhenium University of Shanghai for Science and Technology Shanghai China; ^2^ Department of Cardio‐Pulmonary Circulation, School of Medicine Shanghai Pulmonary Hospital, Tongji University Shanghai China; ^3^ Respiratory Medicine Dongchuan District People's Hospital Kunming China

**Keywords:** cardiovascular diseases, endothelial cells, prolactin, vasoinhibins

## Abstract

Prolactin (PRL) is a polypeptide hormone that is mainly synthesized and secreted by the lactotroph cells of the pituitary. There are two main isoforms of PRL: 23‐kDa PRL (named full‐length PRL) and vasoinhibins (including 5.6–18 kDa fragments). Both act as circulating hormones and cytokines to stimulate or inhibit vascular formation at different stages and neovascularization, including endothelial cell proliferation and migration, protease production, and apoptosis. However, their effects on vascular function and cardiovascular diseases are different or even contrary. In addition to the structure, secretion regulation, and signal transduction of PRL/vasoinhibins, this review focuses on the pathological mechanism and clinical significance of PRL/vasoinhibins in cardiovascular diseases.

## Graphical abstract

Vasoinhibins are produced from proteolytic cleavage of 23 kDa PRL by several endogenous proteolytic enzymes, such as cathepsin D, matrix metalloproteinase (MMP), and bome morphogenetic protein‐1 (BMP‐1).35 And vasoinhibins contain the NH2‐terminal part of the mature PRL protein, but not the COOH‐terminal fragment. PRL can promote angiogenesis, while vasoinhibins have the anti‐angiogenic property. The specific impact on vascular is shown in the figure. The solid line has an arrow to indicate the promoting effect, and the other line indicates the inhibiting effect.

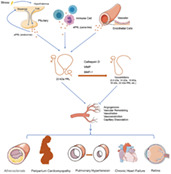

## 1 INTRODUCTION

Prolactin (PRL) is a classical pituitary hormone mainly synthesized and secreted by lactotroph cells of the pituitary.^1^ As a pleiotropic protein, PRL acts as both circulating hormones and cytokines on many physiological processes in lactation, reproduction, osmoregulation, immune response, brain function, metabolism, and angiogenesis.^1^ The full‐length PRL (23 kDa) promotes angiogenesis, but after proteolytic cleavage, the concomitant peptide fragment, as another isoform of PRL (5.6–18 kDa, also called vasoinhibins), acquires antiangiogenic properties.^2^ Generally, the balance or the interactions between full‐length PRL and vasoinhibins regulate vascular functions.^3^, ^4^ PRL/vasoinhibins and vascular function have been reviewed and reported, but most of them focus on the impact on vascular and related signal transduction mechanisms. At present, there is no summary of the molecular structure and disease correlation of PRL/vasoinhibins. In the past decade, accumulating evidence showed the roles of PRL in cardiovascular diseases.^4^, ^5^ For instance, PRL levels are positively associated with all‐cause mortality in cardiovascular diseases^6^; 16‐kDa PRL induces myocardial damage and is involved in the pathogenesis of peripartum cardiomyopathy (PPCM).^7^ PRL and its isoforms have vascular regulation functions, yet our appreciation of the effects of such hormones on cardiovascular health is limited. In this review, we aimed to summarize the structure, secretion regulation, signal transduction of PRL/vasoinhibins, and their pathological mechanism in vascular remodeling. This review will enable researchers to better understand the role of PRL/vasoinhibins in cardiovascular diseases.

## 2 MOLECULAR FORMS OF PRL

**FIGURE 1:**
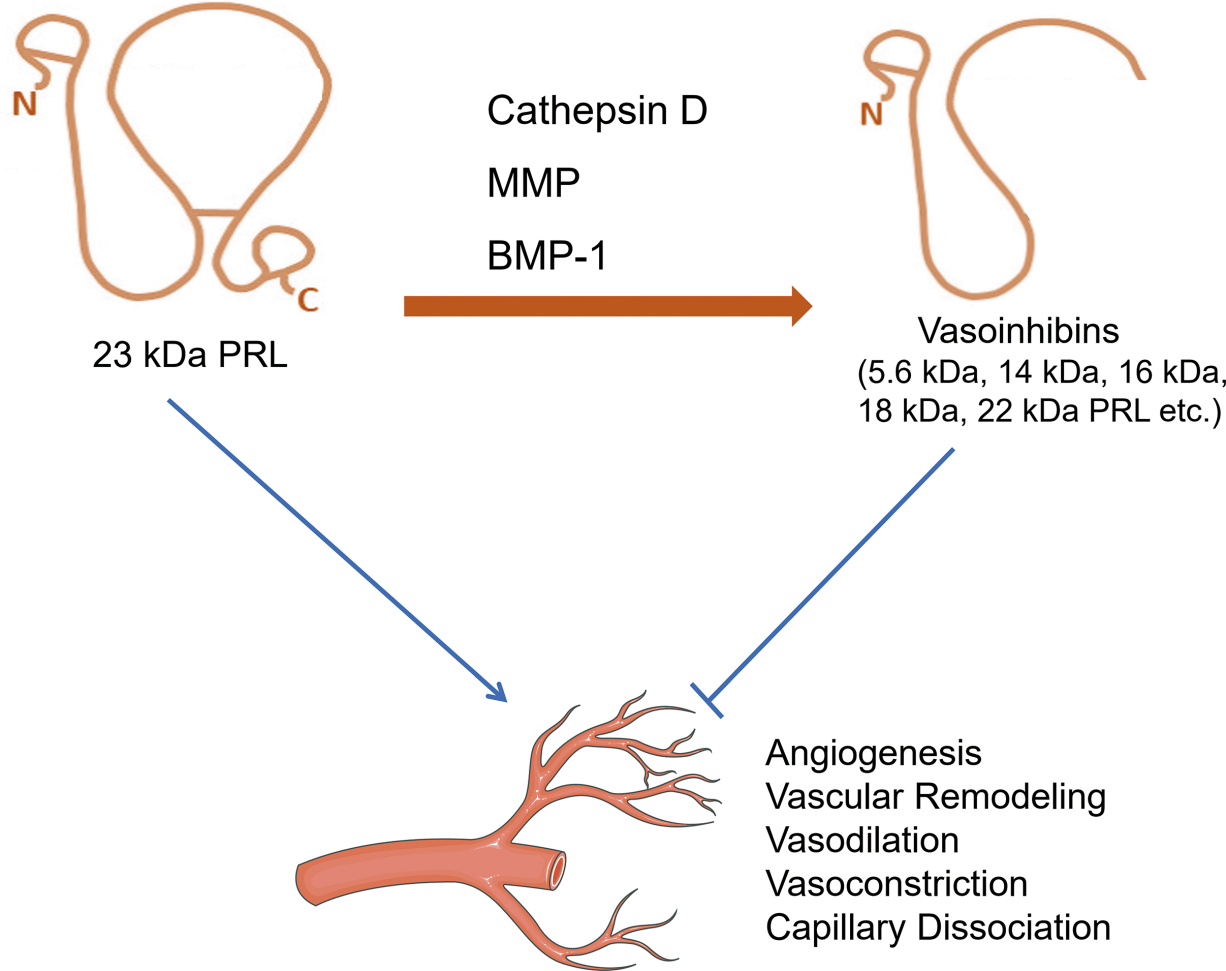
Vasoinhibins are produced from proteolytic cleavage of 23‐kDa PRL by several endogenous proteolytic enzymes, such as cathepsin D, matrix metalloproteinase (MMP), and bone morphogenetic protein‐1 (BMP‐1).^35^ And vasoinhibins contain the NH_2_‐terminal part of the mature PRL protein but not the COOH‐terminal fragment. Prolactin (PRL) promotes angiogenesis, whereas vasoinhibins possess antiangiogenic property. The specific impact on vascular is shown in the figure. The solid line has an arrow to indicate the promoting effect, and the other line indicates the inhibiting effect.

The PRL gene is unique and located on chromosome 6 in the human genome,^8^ containing four introns and five exons and an additional noncoding exon 1a. Transcription of the PRL is regulated by two independent promoter regions. The proximal promoter directs pituitary PRL (pPRL) expression,^9^ whereas the distal promoter with a 5000‐bp upstream of transcription starting site is responsible for extrapituitary PRL (ePRL) mRNA expression.^9^ After cleavage of the 28 amino acid signal peptides, the mature protein containing 199 residues is depicted as the 23‐kDa PRL monomer. In addition, numerous variants of PRL have been identified, including big PRL (dimer of the monomeric form), big‐big PRL (complexes of monomeric form and IgG autoantibodies), and some variants with smaller molecular weight (14, 16, and 22‐kDa). As shown in Figure 1, a number of variants with a molecular weight between 5.6^10^ and 18 kDa are defined as a novel family^11^ and named vasoinhibins, as these peptides share blood vessel inhibitory properties. Vasoinhibins are derived from proteolytic cleavage of the full‐length PRL near or within the long loop that connects the third and fourth α helices,^8^, ^12^ so they contain the NH_2_‐terminal part of the mature PRL protein instead of the COOH‐terminal fragment.

## 3 SECRETION AND REGULATION OF PRL

**FIGURE 2:**
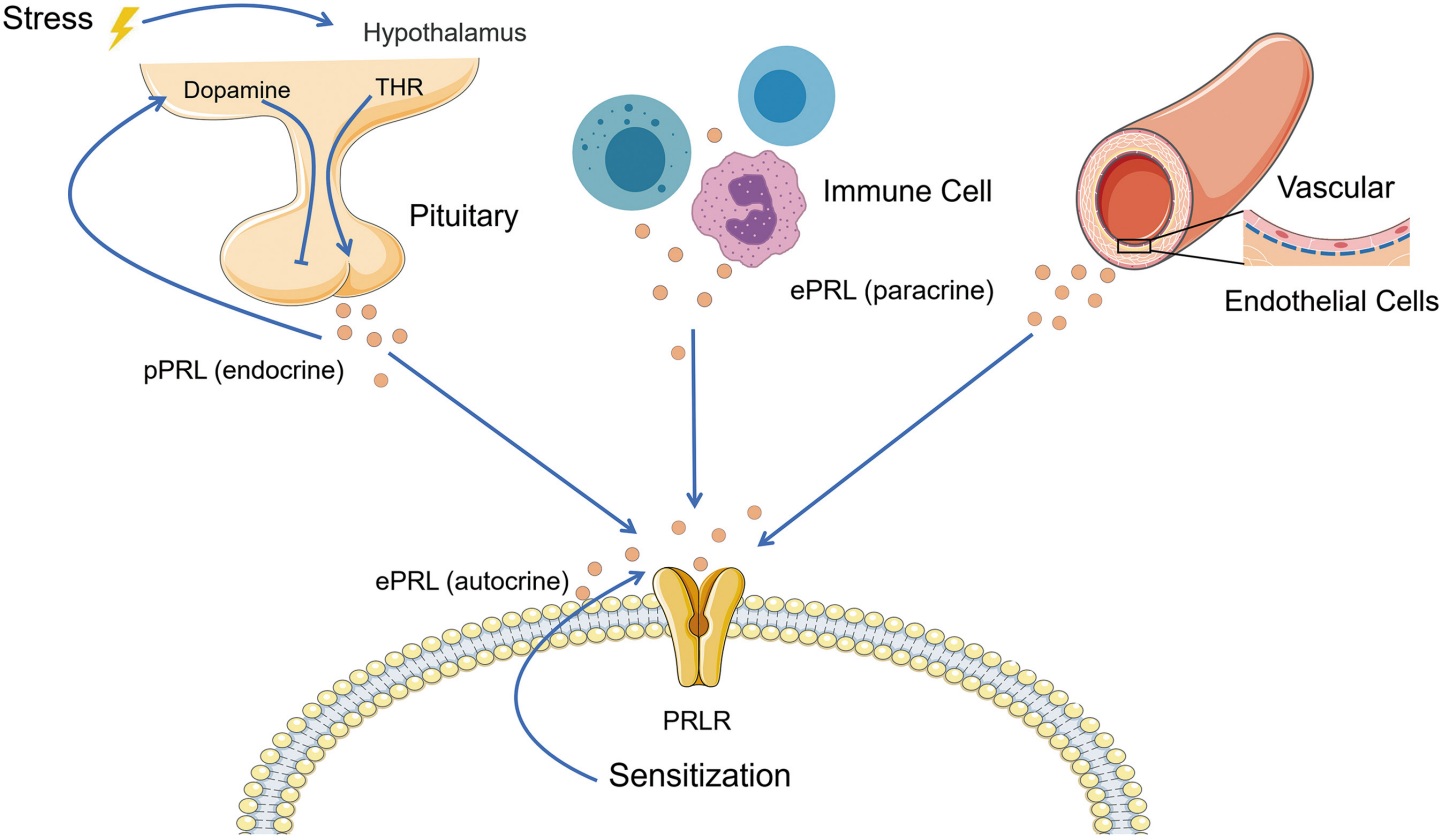
pPRL (pituitary prolactin) is mainly synthesized and secreted by the lactotroph cells of the anterior pituitary, and this process is mainly regulated by dopamine and thyroid stimulating hormone releasing hormone (TRH) released in the hypothalamus. pPRL exerts a negative feedback effect on its own secretion by affecting dopamine secretion in the hypothalamous or direct action on lactotroph cells. PRL is also produced by autocrine and paracrine cells in numerous extrapituitary tissues (ePRL), such as immune cells and vascular endothelial cells.^85^ Promotion is indicated by a solid line with arrow, and the other line indicates inhibition.

As shown in Figure 2, pPRL is mainly synthesized and secreted by the lactotroph cells of the anterior pituitary,^8^, ^9^ and this process is regulated by a number of prolactin‐releasing factors (PRFs) and prolactin‐inhibiting factors (PIFs)^13^ released from the hypothalamus. The PIFs include dopamine, gonadotropin‐combined peptide, and melanocyte stimulating hormone.^14^, ^15^ PRFs include thyroid stimulating hormone releasing hormone (TRH), gonadotropin‐releasing hormone (GnRH), angiotensin II, and vasoactive peptide.^16^ As the most important PIFs, dopamine inhibits the secretion of pPRL via the type 2 dopamine receptor on the surface of lactotroph cells.^17^ And pPRL exerts a negative feedback effect on its own secretion by promoting dopamine secretion in the hypothalamous^17^ or in an autocrine manner.^1^

Of note, PRL is also produced by cells in numerous extrapituitary tissues (ePRL), including endothelial cells (ECs),^18^ fibroblasts,^19^ and neuronal and immune cells.^20^ The regulation of ePRL is dissimilar to that of pPRL and is typically cell‐ or tissue specific. In decidua, the expression of PRL is controlled by many cytokines (IFNγ and IL‐2), transcription factors (Ets‐1), and signaling peptides (cAMP and protein kinase A) that act either via well‐defined regulatory pathways or by direct binding to putative control elements within the PRL promoter regions.^20^ In peripheral blood mononuclear cells, PRL levels can be regulated by calcitriol (the hormonal form of vitamin D).^20^ Very little is known about ePRL regulation in the vasculature. Previous study showed that PRL expressed in ECs could act in an autocrine manner to regulate cell proliferation.^21^ In addition, STAT5/PRL/vascular endothelial growth factor (VEGF) signaling cascade was proven to exist in human brain ECs and implicate PRL and VEGF as autocrine regulators of EC migration, invasion, and tube formation.^22^

Pituitary vasoinhibin generation is closely intertwined with PLR production, as vasoinhibins are produced from proteolytic cleavage of 23‐kDa PRL by several endogenous proteolytic enzymes, such as cathepsin D,^12^ matrix metalloproteinase (MMP),^23^ and bone morphogenetic protein‐1.^24^ However, the ratio of vasoinhibin generation to PRL synthesis is not fixed; instead, it varies under physiological control. Previous studies in rodents and humans revealed that the ratio of pituitary vasoinhibin to PRL can increase from 0.22 to 0.77 after pregnancy and to 0.99 after perphenazine treatment, a dopamine D1 and D2 receptor antagonist.^11^ The ratio is also increased by treatment with estrogen and decreased by TRH.^11^ In addition, oxidative stress increases the activity of cathepsin D to cleave PRL,^25^ whereas hypoxia decreases cathepsin D–induced vasoinhibin generation.^26^ Except for the pituitary, vasoinhibins can be produced in other tissues, including the human endothelium, the placenta, the cartilage, the retina, and the heart.^9^ At the local tissue, the vasoinhibin levels are under the regulation of both utilization of circulating and locally produced PRL and the level of activity of local PRL cleaving enzymes,^11^ indicating that the microenvironment is important in the regulation of local isoforms of PRL.^11^

## 4 PRL RECEPTOR AND SIGNALIZATION

**FIGURE 3:**
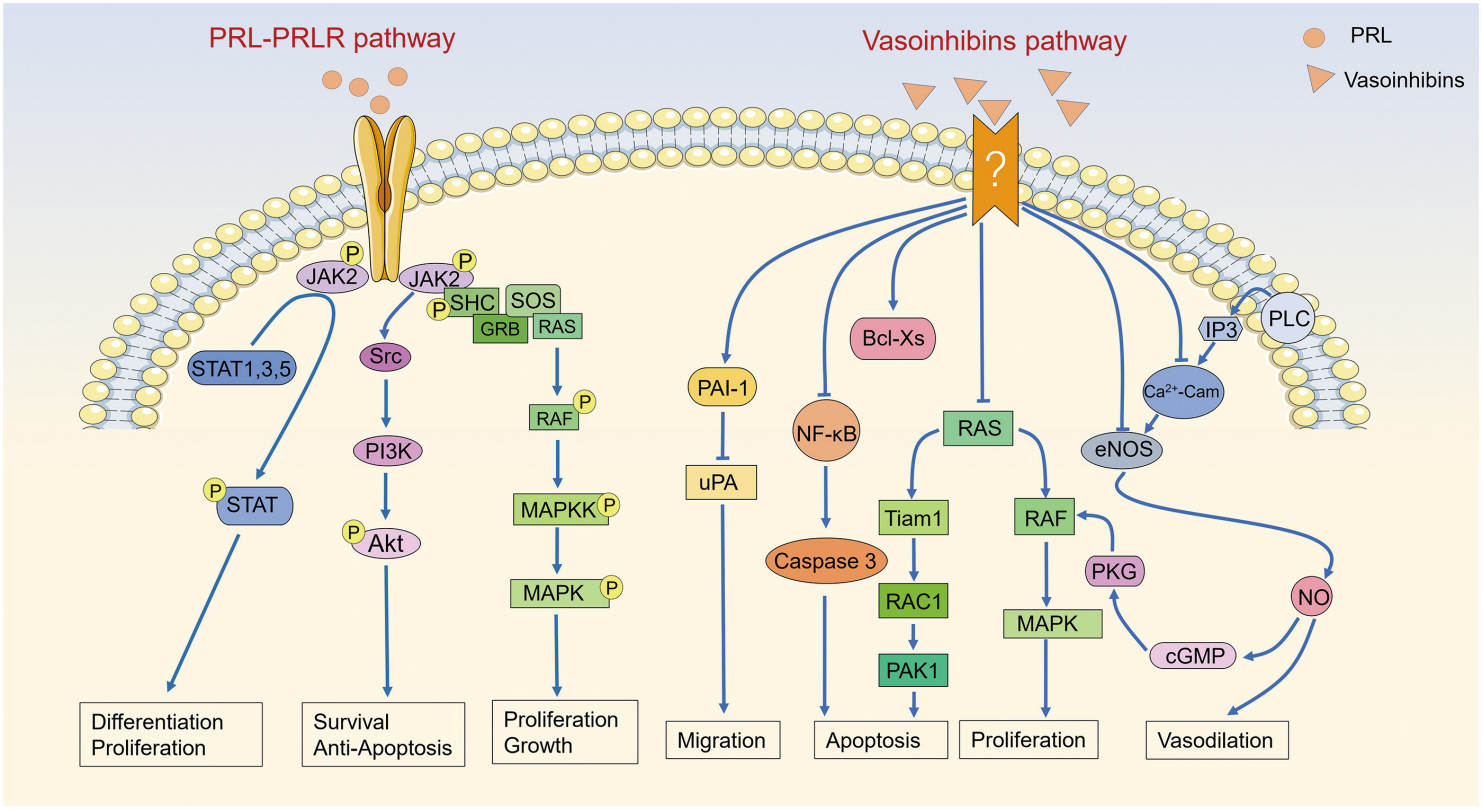
Signal pathways that may be involved in the combination of PRLR and PRL (prolactin): canonical Janus kinase 2 (JAK2)‐signal transducer and activator of transcription (STAT) pathway, mitogen‐activated protein kinase (MAPK) pathway, and phosphatidylinositol‐3‐kinase (PI3K)/Akt pathways. Signal transduction pathways are known to be activated in endotheliall cells by vasoinhibins.^39^ The binding of vasoinhibins to its receptor and the recognition and localization of vasoinhibins are unknown, including increasing the expression of PAI‐1, activating Bcl Xs and/or NFkB, and blocking the stimulating effect of vascular endothelial growth factor (VEGF) on eNOS, RAS–MAPK pathway, or RAS–PAK1 pathway. Promotion is indicated by a solid line with arrow, and the other line indicates inhibition. IP3, inositol trisphosphate; PAI‐1, plasminogen activator inhibitor‐1; PLC, phospholipase C; u‐PA, urokinase‐type plasminogen activator.

PRL activities are normally mediated by its specific highly affinitive receptor (PRLR), which is expressed in many tissues, especially the liver, breast, adrenal gland, and hypothalamus.^27^ The PRLR is a member of the hematopoietic cytokine receptor superfamily and encoded by a gene located on chromosome 5p13‐14. PRLR consists of an extracellular domain that binds PRL, a single transmembrane domain, and a cytoplasmic domain.^28^ The main isoform found in humans is a long near‐ubiquitous 598 amino acid protein and has a mass of 90 kDa. Due to alternative splicing, there are several different isoforms of PRLR, including short forms, that lack the cytoplasmic domain and predominate in ECs of micro‐ and macrovascular origins.^29^ PRLR can bind at least three ligands, 23‐kDa PRL, placental lactogen, and growth hormone, as they are all classical pituitary hormones, structurally, corresponding to a long‐chain class‐I helical cytokine.^8^ When PRL binds to this long isoform of PRLR, several intracellular signaling pathways are activated, including JAK2/STAT (Janus kinase 2/signal transducer and activator of transcription),^8^, ^30^ Ras/Raf/MAPK (mitogen‐activated protein kinase),^10^, ^31^ and PI3K/Akt (phosphoinositide 3‐kinase/protein kinase B)^32^ (Figure 3). Jak2 is a nonreceptor tyrosine kinase that is rapidly active (within 30–60 s) after PRL stimulation, resulting in STAT phosphorylation (STAT1, STAT3, and STAT5) and downstream gene expression, such as VEGF, to induce migration, invasion, and tube formation of ECs.^22^ The MAPK pathway is another important cascade activated by PRL and involves the SHC/GRB2/SOS/RAS/RAF intermediaries upstream of MAPK kinases.^1^

Due to the unique structure, vasoinhibins lose the ability to bind PRLR and cannot activate similar intracellular signaling pathways as the full‐length PRL.^1^ A specific, high‐affinity, saturable binding site was reported on the membranes of capillary ECs decades ago, although its structure has not been identified yet.^33^ And then, Khalid Bajou^34^ identified plasminogen activator inhibitor‐1 (PAI‐1) as a binding partner of 16‐kDa PRL, and a multicomponent complex formed by PAI‐1, urokinase, and the urokinase receptor is required for the full antiangiogenic activity of 16‐kDa PRL on ECs. These two binding partners or receptors mediate the vasoinhibin blockage of various signaling pathways, such as Ras–Raf–MAPK, Ras–Tiam1–Pak1, and PLCγ–IP3–eNOS.^11^, ^35^

## 5 PRL IN REGULATION OF VASCULAR FUNCTIONS

The vascular actions and signaling mechanisms of PRL and vasoinhibins have been discussed in various reviews.^1^, ^35^, ^36^ In summary, circulating or local PRL acts on ECs, immune cells, fibroblasts, pericytes, and smooth muscle cells in a paracrine/autocrine manner, thereby stimulating or inhibiting the proliferation, dilation, permeability, and regression of blood vessels.^5^ These opposite effects exist as the full‐length PRL (23 kDa) and promote angiogenesis,^10^ but the proteolytic isoforms of PRL, vasoinhibins, acquire antiangiogenic properties.^23^, ^28^ In general, ECs (the main vascular intimal cells), smooth muscle cells (the typical cell type of arterial medial membrane), fibroblasts (the typical adventitial cells), pericytes (important vascular cells), and immune cells (perivascular cells) play an important role in vascular remodeling. Here, we summarize the effects of PRL/vasoinhibins on vascular function by stimulating different types of cells.

### 5.1 Endothelial cells

ECs line the inner surface of vessels to support tissue growth and repair. Apoptosis by EC injury is usually considered as the initiating factor of vascular remodeling, and its excessive proliferation and apoptosis resistance lead to vascular intimal thickening, lumen stenosis, and even occlusion. PRL was reported to promote cell migration, invasion, and tube formation in ECs through JAK2‐STAT5 pathway^22^ and to decrease vasopermeability by upregulating the expression of tight‐junction proteins between ECs.^37^ Meanwhile, PRL stimulates the expression of proangiogenic factors by activating various non‐ECs, such as fibroblast growth factor‐2 (FGF‐2) and VEGF. In addition, heme‐oxygenase‐1 is confirmed to be the second messenger for PRL‐mediated angiogenesis and EC proliferation.^18^ Therefore, all the findings suggest that PRL acts not only as a systemic but also as an autocrine/paracrine positive regulator of angiogenesis.^11^, ^35^

On the contrary, vasoinhibins inhibit angiogenesis, vasodilation, and vasopermeability by inhibiting the action of several vasoactive substances, such as VEGF, FGF‐2, and interleukin 1β (IL‐1β), via the Ras–Raf–MAPK pathway and the Ras–Tiam1–Rac1–Pak1 pathway, as well as microRNA‐146a^38^ on ECs. In addition to these pathways, vasoinhibins may block the mechanism of eNOS activation by interfering with intracellular Ca^2+^ calmodulin binding, blocking acetylcholine and bradykinin‐induced Ca^2+^ transients in ECs.^39^ Besides, vasoinhibins can act as a potent pro‐inflammatory cytokine that stimulates iNOS expression and NO production, and exogenous NO can reverse the inhibition of vasoinhibins on VEGF‐induced EC proliferation.^19^ Subsequently, NO stimulates cGMP production and activates cGMP‐dependent protein kinase (PKG), thus leading ultimately to the activation of Raf–MAPK signaling.^39^ However, the effects of PRL on the angiogenic process may be much more complex. Although PRL can promote angiogenesis at different anatomical sites in the body, the other mediators in the milieu of specific tissue or organ could affect the pro‐angiogetic feature of PRL. For example, PRL‐mediated proliferation may not occur when PRLR in the vascular endothelium is already occupied by locally produced hormones.^18^ The aforementioned signal pathway is shown in Figure 3.

### 5.2 Immune cells

Immune cells are an important location for PRL production; they participate in angiogenesis by producing and releasing a large number of proangiogenic mediators.^36^ Simultaneously, they also have antiangiogenic properties in specific diseases (inflammation and tumor). Research has confirmed PRL/vasoinhibins could act indirectly by recruiting immune cells to produce other regulators.^40^, ^41^ Pável Montes de Oca et al. demonstrated that PRL promotes the adhesion of peripheral blood mononuclear cells to human umbilical vein ECs by activation of integrins (CD11a/CD18 and CD49d/CD29), as well as selective recruitment of T cells via upregulating CXCR3, and then induces transendothelial leukocyte migration and stimulates production of chemokines in the local microenvironment.^40^ And PRL has recently been considered as a local regulator of macrophage responses. Macrophages treated with PRL showed enhanced expression of heme oxygenase‐1 and enhanced release of VEGF, which controlled the angiogenesis induced by macrophage PRL.^42^ Furthermore, PRL/vasoinhibins can directly induce a pro‐inflammatory/anti‐inflammatory response by affecting the infiltration of lymphocytes, macrophages, and neutrophils.^43^ In rheumatoid arthritis (RA), pro‐inflammatory cytokines and PRL stimulate RA synovial cells to produce MMP. MMP cleaves PRL into vasoinhibin, which can suppress the neovascularization required for pannus formation.^36^

### 5.3 Fibroblasts

Fibroblasts are the main cells of vascular adventitia. Their proliferation is involved in the formation of neointima and leads to pathological vascular remodeling.^44^ It has been reported that PRL/vasoinhibins can stimulate fibroblasts and affect vascular function. First, vasoinhibins can stimulate the active nuclear factor‐κB (NF‐κB) signaling pathway in fibroblasts,^45^ which are the main mesenchymal cells that produce the amount of extracellular matrix in tissue repair and vascular remodeling.^44^ Then, vasoinhibins inhibit the survival of ECs and fibroblasts^35^ by activating proapoptotic proteins of the Bcl‐2 family and the NF‐κB‐mediated caspases.^5^, ^46^ Besides, in RA synovial tissue, PRL and PRL‐like polypeptides could participate in a bidirectional communication between immunocytes and fibroblasts and might act via proto‐oncogenes and transcriptional factors, leading to cell proliferation, that is, neo‐angiogenesis, and the production of catabolic enzymes such as MMPs and cathepsins.^47^

### 5.4 Pericytes

Pericytes embed in the basement membrane of capillary ECs, communicate with ECs through physical contact and paracrine signals, and monitor and stabilize the maturation process of ECs. As integral constituents of blood vessels, pericytes are essential regulators of vascular development, stabilization, maturation, and remodeling^44^; 16‐kDa hPRL (one form of vasoinhibins) can inhibit the migration of pericytes to ECs by perturbing Dll4/Notch4 crosstalk and EphrinB2 expression to restrict tumor vessel maturation.^41^ Furthermore, it has been demonstrated that 16‐kDa hPRL affects vessel maturation by inhibiting the outgrowth of a pericyte/smooth muscle cell network (in an aortic ring assay) and pericyte recruitment via PDGF‐B/PDGFR‐B, Ang/Tie2, and Delta/Notch pathways.^41^

### 5.5 Smooth muscle cells

Smooth muscle cells, a major structural component of the vessel wall, provide the main support for the structure of the vessel wall and regulate vascular tone to maintain intravascular pressure and tissue perfusion.^48^ Sauro and Zorn^49^ found that PRL‐induced aortic smooth muscle cell proliferation is mediated through PKC pathway, suggesting the role of PRL in vascular smooth muscle cell hyperplasia and the pathogenesis of cardiovascular diseases such as hypertension and atherosclerosis. However, the research on PRL affecting vascular function by stimulating smooth muscle cells is insufficient, which needs to be further explored.

## 6 PHYSIOLOGICAL AND CLINICAL IMPLICATIONS OF PRL IN CARDIOVASCULAR DISEASES

A large amount of evidence showed that there is a causal relationship between PRL and cardiovascular diseases,^50^, ^51^ which is summarized in Table 1. The roles of PRL in cardiovascular disorders are discussed in the following section.

**TABLE 1:** Summary of the clinical reported studies on the relationship between cardiovascular diseases and PRL

Author	Number/age/female (%)	Follow‐up time	Main outcome measures	Results
Year				
Anne Q. Reuwer^86^	3375	7 years	Biochemical analysis, BMI, systolic blood pressure, etc.	PRL may be related to atherosclerotic plaque
2009	45–79 years			
	Not mentioned			
Xiao Bing Jiang^87^	91	Not mentioned	BMI, FMD, IMT, inflammatory markers, serum glucose, insulin, lipid, and apolipoprotein profiles	Hyperprolactinemia may be involved in preclinical increase in carotid IMT, directly or by promoting atherogenic factors.
2014	Not mentioned			
	Not mentioned			
Robin Haring^6^	3929	10.1 years	BMI, serum PRL analysis, mortality follow‐up, LDL measurement, etc.	Positive association of PRL with all‐cause and cardiovascular mortality
2014	20–81 years			
	1983 (50.5%)			
Sanja Bekić^88^	92	Not mentioned	BMI, cardiovascular risk factors, PRL measurement, etc.	There were relationships between serum PRL and various cardiovascular risk factors
2018	50–89 years			
	58 (63%)			
G. Georgiopoulos^89^	201	3 years	Circulating PRL levels, FMD, PWV, BP, etc.	PRL levels predicted accelerated arterial stiffening
2017	After menopause			
	201 (100%)			
G. A. Georgiopoulos^54^	76	3 months	Biochemical analysis, IMT, PWV, BP, laser doppler fluximetry, etc.	Prolactin may affect central/peripheral blood pressure and arterial stiffness in early menopause
2009	54.4 ± 4.9			
	76 (100%)			
Juan Jesús Carrero^90^	457/173	Not mentioned	Biochemical measurements, BP, MAP, etc.	Cardiovascular event risk and mortality were associated with PRL in CKD patients
2012	52 ± 12/65 ± 12 years			
	228 (50%)/62 (36%)			
Denise Hilfiker‐Kleiner^60^	63	6 months	RVEF, LVEF, recovery rate of left ventricular function, echocardiography, etc.	Bromocriptine was associated with low morbidity and mortality in PPCM patients
2017	Postpartum patients			
	63 (100%)			
Kate E. Therkelsen^91^	3232	6.1 years	BMI, MDCT, PRL assessment, CVD risk factor assessment, etc.	PRL levels were associated with low HDL cholesterol and are associated with the incidence rate of hypertension in men
2016	Mean: 40.4 years			
	1684 (52.1%)			
Uta Hönicke^66^	103	Not mentioned	Serum levels of PRL and 16‐kDa PRL, 6‐MWT, VO_2_max, echocardiography, etc.	PRL and 16‐kDa PRL were significantly higher in PH and were inversely correlated with 6‐MWT and VO2max
2012	18–80 years			
	Not mentioned			
John T. Parissis^82^	180	8 months	Serum levels of PRL, 6‐MWT, LVEF, SDS, etc.	Serum PRL was associated with depressive symptoms and was an independent predictor of prognosis in advanced chronic heart failure
2013	65 ± 12 years			
	Not mentioned			
Robin Haring^55^	804	5 years	Echocardiography, LVH, LVM, RWT, PRL concentration, etc.	There were inverse associations between PRL and LVM change, incident LVH, and altered LV geometry in men but not in women
2012	>45 years			
	441 (55%)			
Alireza Amirzadegan^92^	414	Not mentioned	Serum PRL levels, coronary angiography, Gensini score, etc.	No correlation between serum PRL levels and coronary atherosclerosis
2019	After menopause			
	414 (100%)			
A. O. Koca^93^	109	1 year	Serum PRL levels, periphery, and central tension measurement, arterial stiffness measurements, etc.	Mildly elevated PRL levels in the young population do not increase arterial stiffness, but the long‐term effects are not known
2021	18–60 years			
	Not mentioned			

Abbreviations: 6‐MWT, 6‐minute‐walk test; BMI, body mass index; BP, blood pressure; CKD, chronic kidney disease; CVD, cardiovascular disease; FMD, flow‐mediated dilation; HDL, high‐density lipoprotein; IMT, intima‐media thickness; LDL, low‐density lipoprotein; LVEF, left ventricular ejection fraction; LVH, left ventricular hypertrophy; LVM, left ventricular mass; MAP, mean arterial pressure; MDCT, multidetector computed tomography; PH, pulmonary hypertension; PPCM, peripartum cardiomyopathy; PRL, prolactin; PWV, pulse‐wave velocity; RVEF, right ventricular ejection fraction; RWT, relative wall thickness; SDS, Zung self‐rated depression scale; VO_2_max, peak oxygen uptake.

### 6.1 Arteriosclerosis

Atherosclerosis is a chronic inflammatory disease; unstable atherosclerotic plaque rupture, vascular stenosis, or occlusion caused by platelet aggregation and thrombosis lead to acute cardiovascular diseases.^52^ Several studies suggested hyperprolactinemia contributes to the development of atherosclerosis.^53^ In menopausal women, PRL, even at normal levels, was found to positively correlate with blood pressure (BP), arterial stiffness, and the Heart Score of the European Society of Cardiology (a composite index that predicts mortality within 10 years).^54^ However, inverse associations between PRL and left ventricular mass change, incident left ventricular hypertrophy, and altered left ventricular (LV) geometry were observed in men rather than in women.^55^ Besides, patients with hyperprolactinemia have significant increases in mean carotid intima thickness, capillary blood glucose, insulin resistance, and hypersensitive C‐reactive protein.^56^ Consistent with these observations, a prospective cohort study found higher daytime PRL levels are an independent predictor of increased risk of future hypertension in postmenopausal women.^56^ Robin et al. evaluated 3929 individuals aged 20–81 years from the population‐based Study of Health in Pomerania and observed a positive association between PRL concentrations and all‐cause and cardiovascular mortality by age‐ and multivariable‐adjusted Cox regression models.^6^

In addition to clinical studies, the pathological mechanism of PRL affecting cardiovascular diseases has been explored in in vivo and in vitro studies.^57^ Using transgenic mice, Albert S. Chang et al. confirmed that a threefold increase in plasma PRL could significantly increase BP and markedly impair cardiac function by the activation of eNOS and NO production.^58^ Ronald J. van der Sluis et al. induced long‐term receptor antagonist Del1‐9‐G129R‐hPRL in mice by bone marrow transplantation in irradiated atherosclerosis‐susceptible LDL receptor knockout mice and found blocking PRLR reduced the atherogenic index but had no effect on the initial development of atherosclerotic lesions.^59^ Meanwhile, several in vitro studies indicated a direct effect of PRL on ECs,^57^ smooth muscle cells,^49^ fibroblasts,^34^ and other cells, which eventually may lead to endothelial dysfunction, pathologic vascular tone, arterial stiffening, increased BP, and further end‐organ disease.^35^, ^54^, ^56^

### 6.2 Peripartum cardiomyopathy

PPCM is an idiopathic, multifactor cause of heart failure occurring at the end of pregnancy or in the first months after delivery; 23‐kDa PRL and the production of a cleaved 16‐kDa fragment of PRL have emerged as potential key factors in the pathophysiology of PPCM,^60^ of which 16‐kDa PRL serves as the main trigger of PPCM.^61^ Experimental studies suggested that 23‐kDa PRL promoted inflammation^60^ and 16‐kDa PRL induced severe vascular endothelial damage and subsequent cardiomyocyte dysfunction, ultimately resulting in heart failure in PPCM.^62^ The inhibition of PRL release by bromocriptine, a dopamine D2 receptor agonist, was found to prevent the occurrence of PPCM possibly via the reduced production of 16‐kDa PRL.^62^ In this multicenter trial, PPCM patients were assigned to short‐term or long‐term bromocriptine treatment in addition to standard heart failure therapy. It could be postulated that bromocriptine treatment could decrease the level of 23 kDa in patients, thus reducing the adverse effects of 16 kDa produced from ECs and cardiomyocytes. The result showed bromocriptine treatment was associated with a high rate of full left ventricle recovery and low morbidity and mortality in PPCM patients compared with other PPCM cohorts in the absence of bromocriptine treatment,^60^ and even 1 week more on bromocriptine treatment to standard heart failure was already beneficial. In another clinical experiment, the results also showed that patients with PPCM treated with bromocriptine had greater recovery of left ventricular ejection fraction and reduced mortality.^63^

In PPCM mice, a cardiomyocyte‐specific knockout for STAT3 (conditional knockout [CKO]), cardiac PAI‐1 as a binding partner of 16‐kDa PRL, its expression was higher than in postpartum wild‐type controls, whereas a systemic PAI‐1‐knockout in CKO mice accelerated peripartum cardiac fibrosis, inflammation, heart failure, and mortality.^64^ Furthermore, researchers found that 16‐kDa PRL induced miR‐146a expression in ECs, which attenuated angiogenesis through downregulation of NRAS.^38^ CKO mice displayed increased cardiac miR‐146a expression with coincident downregulation of Erbb4, NRAS, Notch1, and Irak1.^38^ Blocking miR‐146a with locked nucleic acids or antago‐miRs attenuated PPCM in CKO mice without interrupting 23‐kDa PRL signaling.^38^

### 6.3 Pulmonary hypertension

Pulmonary hypertension (PH) is a lethal and progressive cardiovascular disorder characterized by pulmonary vascular remodeling, resulting in increased pulmonary artery pressure and progressive right ventricular dysfunction.^65^ A previous study showed that serum levels of PRL and 16‐kDa PRL were significantly higher in patients with precapillary PH, which was negatively correlated with 6‐minute‐walk test and peak oxygen uptake.^66^ A case report described a patient presented with PH after several years' treatment for hyperprolactinemia with cabergoline and bromocriptine.^67^ These results indicated that PRL and 16‐kDa PRL might contribute to the pathophysiology of PH,^66^, ^67^ although the exact impact of PRL on PH is unknown. Endothelial dysfunction is a major player in developing PH, and tremendous evidence provided earlier has demonstrated that PRL/vasoinhibins regulate EC function.^2^, ^38^, ^46^ Additional important factors involved in the pathogenesis on PH include genetic predisposition,^68^, ^69^ autoimmunity,^65^ epigenetic regulation,^70^, ^71^ inflammation,^72^, ^73^ metabolic derangement,^74^, ^75^ and environment insults.^76^, ^77^ PRL has been shown to stimulate the immune cells, increase the synthesis of immunoglobulins and autoantibodies, and promote aberrant immune responses.^78^ The development of several autoimmune diseases associated with PH is also strongly influenced by PRL,^78^ such as systemic lupus erythematosus and systemic sclerosis. It indicates that PRL can act as immune stimulants to induce PH. Furthermore, PRL enhances the release of several pro‐inflammatory cytokines, such as IL‐1β, tumor necrosis factor alpha (TNF‐α), IL‐12, and IFNγ, and these can mediate pulmonary vascular remodeling.^72^, ^73^, ^79^ Besides, PRL has been reported to affect cellular glycolysis^80^ and protect cells from hypoxia‐induced damage,^81^ which might also exist in PH development, although currently no evidence has directly demonstrated the role of PRL/vasoinhibins on the pathophysiology of PH.

### 6.4 Chronic heart failure

Heart failure encompasses heart dysfunction due to any type of cardiovascular diseases. Serum PRL levels have been shown to have a significantly negative correlation with left ventricular ejection fraction (LVEF), 6‐minute‐walk test, and natriuretic peptides in patients with chronic heart failure, and patients who had higher baseline PRL levels were at high risk of death or hospitalization.^82^ In this study, the author also found that PRL levels were significantly associated with the pro‐inflammatory markers IL‐6 and TNF‐α and the anti‐inflammatory cytokine IL‐10 and proposed that a vicious combination of PRL, oxidative stress, and inflammation may attribute to the pathogenesis of chronic heart failure.^82^ However, whether prolactin elevation is a causal factor of chronic heart failure remains to be elucidated.

### 6.5 Retinopathy of prematurity

Retinopathy of prematurity (ROP) is a potentially blinding retinal neovascularization disease. In a prospective, case–control study, serum PRL and vasoinhibins were measured weekly in 90 preterm infants diagnosed with ROP or controls between 1 and 9 weeks after birth. PRL levels were found to be higher in ROP patients than in controls during the first (vasoinhibitory) and the second (vasoproliferative) phases of ROP.^83^ Although vasoinhibin levels (combined with 14‐ and 16‐kDa PRL) significantly increased during the first week after birth in ROP patients, the levels became equal to those in controls during the postnatal weeks, which indicated that dysregulation of the PRL/vasoinhibin axis exerted vasoinhibitory property at early stage and vasoproliferative feature in late phases of the disease. Besides, there was a correlation between PRL in aqueous humor and subretinal fluid.^84^ The 16‐kDa PRL isoform was more concentrated in subretinal fluid than in serum and was generated from PRL by subretinal fluid proteases; 16‐kDa PRL derived from PRL internalized from the circulation or synthesized intraocularly can stimulate apoptosis‐induced vascular regression.^84^

## 7 CONCLUDING REMARKS AND FUTURE DIRECTIONS

This review focused on the specific effects of the PRL hormone family on vascular function, including receptors and signaling pathways, pathological mechanisms, and clinical studies of vascular diseases. Exciting studies have shown that the PRL hormone family has a significant impact on vascular remodeling. However, the extent of these effects on the physiology and pathology of the disease remains to be determined. This paper provided a new idea for research on the PRL family on vascular remodeling. The development of adjuvant therapies to target PRL and/or PRLR with a focus on cardiology opens up new perspectives for this old hormone.

## AUTHOR CONTRIBUTIONS

Hui Zhao, Wenhui Wu, and Yuqing Miao developed the concept. Sugang Gong, Yongcong Shi, and Cijun Luo performed literature review. Hui Zhao, Sugang Gong, Yongcong Shi, and Hongling Qiu wrote the manuscript. Jing He and Yuanyuan Sun edited the paper. Yuxia Huang and Shang Wang drew the figures. All authors contributed to manuscript revision, read, and approved the submitted version.

## CONFLICT OF INTEREST

The authors declare that they have no competing interests.
